# MKL1 mediates TNF-α induced pro-inflammatory transcription by bridging the crosstalk between BRG1 and WDR5

**DOI:** 10.7555/JBR.32.20170025

**Published:** 2017-07-30

**Authors:** Wenping Xu, Quanyi Zhao, Min Wu, Mingming Fang, Yong Xu

**Affiliations:** 1. Department of Medicine, Jiangsu Jiankang Vocational College, Nanjing, Jiangsu 211800, China; 2. Department of Biochemistry and Molecular Biology, College of Life Sciences, Wuhan University, Wuhan, Hubei 430072, China; 3. Department of Pathophysiology, Key Laboratory of Cardiovascular Disease and Molecular Intervention, Nanjing Medical University, Nanjing, Jiangsu 211166, China.

**Keywords:** MKL1, WDR5, BRG1, macrophage, transcriptional regulation

## Abstract

Tumor necrosis factor alpha (TNF-α) is a cytokine that can potently stimulate the synthesis of a range of pro-inflammatory mediators in macrophages. The underlying epigenetic mechanism, however, is underexplored. Here we report that the transcriptional modulator megakaryocytic leukemia 1 (MKL1) is associated with a histone H3K4 methyltransferase activity. Re-ChIP assay suggests that MKL1 interacts with and recruits WDR5, a component of the COMPASS complex responsible for H3K4 methylation, to the promoter regions of pro-inflammatory genes in macrophages treated with TNF-α. WDR5 enhances the ability of MKL1 to stimulate the promoter activities of pro-inflammatory genes. In contrast, silencing of WDR5 attenuates TNF-α induced production of pro-inflammatory mediators and erases the H3K4 methylation from the gene promoters. Of interest, the chromatin remodeling protein BRG1 also plays an essential role in maintaining H3K4 methylation on MKL1 target promoters by interacting with WDR5. MKL1 knockdown disrupts the interaction between BRG1 and WDR5. Together, our data illustrate a role for MKL1 in moderating the crosstalk between BRG1 and WDR5 to activate TNF-α induced pro-inflammatory transcription in macrophages.

## Introduction

Macrophages constitute a major source of inflammation in both health and disease states^[1]^. Macrophage-associated inflammation is generally believed to be a double-edged sword serving both protective and pathogenic roles. When orderly programmed, it helps maintain tissue homeostasis. When aberrantly constructed, it disrupts physiologic integrity and contributes to a host of human diseases such as metabolic syndrome^[2]^, atherosclerosis^[3]^, and cancer^[4^–^5]^. In macrophages, inflammation-related transcriptional programs are dictated by a handful of conserved transcriptional factors (TF) that include NF-κB, AP-1, and STAT^[6]^. The activities of these sequence-specific TFs are modulated by various co-factors, which may either enhance or suppress the inflammatory response. We have previously identified megakaryocytic leukemia 1 (MKL1) as a co-factor for NF-κB^[7]^. MKL1 interacts with NF-κB and stimulates NF-κB-dependent trans-activation of target genes to promote the pathogenesis of pulmonary hypertension and colitis^[8^–^10]^. The mechanism whereby MKL1 mediates NF-κB-dependent pro-inflammatory transcription, however, is not completely defined.

Engagement of the epigenetic machinery represents a paradigm of transcriptional regulation in eukaryotes^[11]^. Typically, transcriptionally active chromatin is marked by methylated histone H3K4 while transcriptional repression is synonymous with methylated H3K9 and H3K27^[12]^. In eukaryotes, histone H3K4 methylation is catalyzed by the COMPASS complex (short for complex proteins associated with SET1)^[13]^. COMPASS operates in a modular mode: whereas catalytic subunits, including MLL1, MLL2, MLL3, MLL4, and SET1 are exchangeable, structural/regulatory subunits, including ASH2, WDR5, WDR82, and RpBP5, are invariable. How individual COMPASS components regulate pro-inflammatory transcription in macrophages remains to be clearly sorted out.

A range of epigenetic factors have been documented to participate in the regulation of pro-inflammatory transcription in macrophages. For instance, brahma-related gene 1, or BRG1, was reported to control temporal production of pro-inflammatory mediators induced by lipopolysaccharide (LPS) and free fatty acids^[14^–^15]^. Several histone methyltransferases, including MLL1^[16]^, MLL4^[17]^, and SET7/9^[18]^, have all been implicated in the regulation of macrophage-derived inflammation by modulating NF-κB activity. Here we report that MKL1 regulates NF-κB-dependent pro-inflammatory transcription by moderating the crosstalk between WDR5 and BRG1.

## Materials and methods

### Cell culture

Human monocytic/macrophage-like cells (THP-1, ATCC) and human embryonic kidney fibroblast cells (HEK293, ATCC) were maintained in DMEM supplemented with 10% FBS. Mouse embryonic fibroblast (MEF) cells were isolated from wild type or MKL1 deficient mice^[19]^. Procedures involving animals were approved by the intramural Ethics Committee on Humane Treatment of Experimental Animals.

### Plasmids, transfection, and reporter assay

Expression constructs for MKL1, WDR5, as well as promoter constructs have been described before^[9]^. siRNAs for MKL1 and WDR5 were purchased from Dharmacon. Transient transfections were performed with Lipofectamine LTX (Invitrogen). Luciferase activities were assayed 24-48 h after transfection using a luciferase reporter assay system (Promega). All experiments were repeated three times.

### ChIP and Re-ChIP assay

ChIP assays were performed essentially as described before^[20^–^21]^. Aliquots of lysates containing 200 μg of nuclear protein were used for each immunoprecipitation reaction with anti-MKL1 (Santa Cruz), anti-BRG1 (Abcam), anti-WDR5 (Bethyl Laboratories), anti-dimethylated H3K4 (Millipore/Upstate), and anti-trimethylated H3K4 (Millipore/Upstate). For re-ChIP, immune complexes were eluted with the elution buffer (1% SDS, 100mmol/L NaCO_3_), diluted with the re-ChIP buffer (1% Triton X-100, 2 mmol/L EDTA, 150 mmol/L NaCl, 20 mmol/L Tris pH 8.1), and subject to immunoprecipitation with a second antibody of interest. All experiments were repeated three times.

### RNA extraction and real-time PCR

RNA was extracted using an RNeasy RNA isolation kit (Qiagen). Reverse transcriptase reactions were performed using a SuperScript First-strand synthesis system (Invitrogen). Real-time PCR reactions were performed on an ABI STEPONE Plus (Life Tech) with primers and Taqman probes purchased from Applied Biosystems. All experiments were repeated three times.

### *In vitro* HMT assay

*In vitro* histone methyltransferase assay was performed as described before^[22]^. Briefly, precipitated immune complex was mixed with histone H3 (Millipore), S-adenosyl methionine (SAM, Sigma), BSA, and MAB buffer (50 mmol/L Tris pH 8.5, 20 mmol/L KCl, 10 mmol/L MgCl_2_, 10 mmol/L β-mercaptoethanol, 250 mmol/L sucrose). After incubation at 37°C overnight, SDS loading buffer was added to stop reactions, and the methylation of histone H3 was determined by Western blotting.

### Statistical analysis

One-way ANOVA with post-hoc Scheffe analyses were performed using an SPSS package. *P* values smaller than 0.05 were considered statistically significant (*).

## Results

### MKL1 is associated with a histone H3K4 methyltransferase activity in macrophages

**Fig.1:**
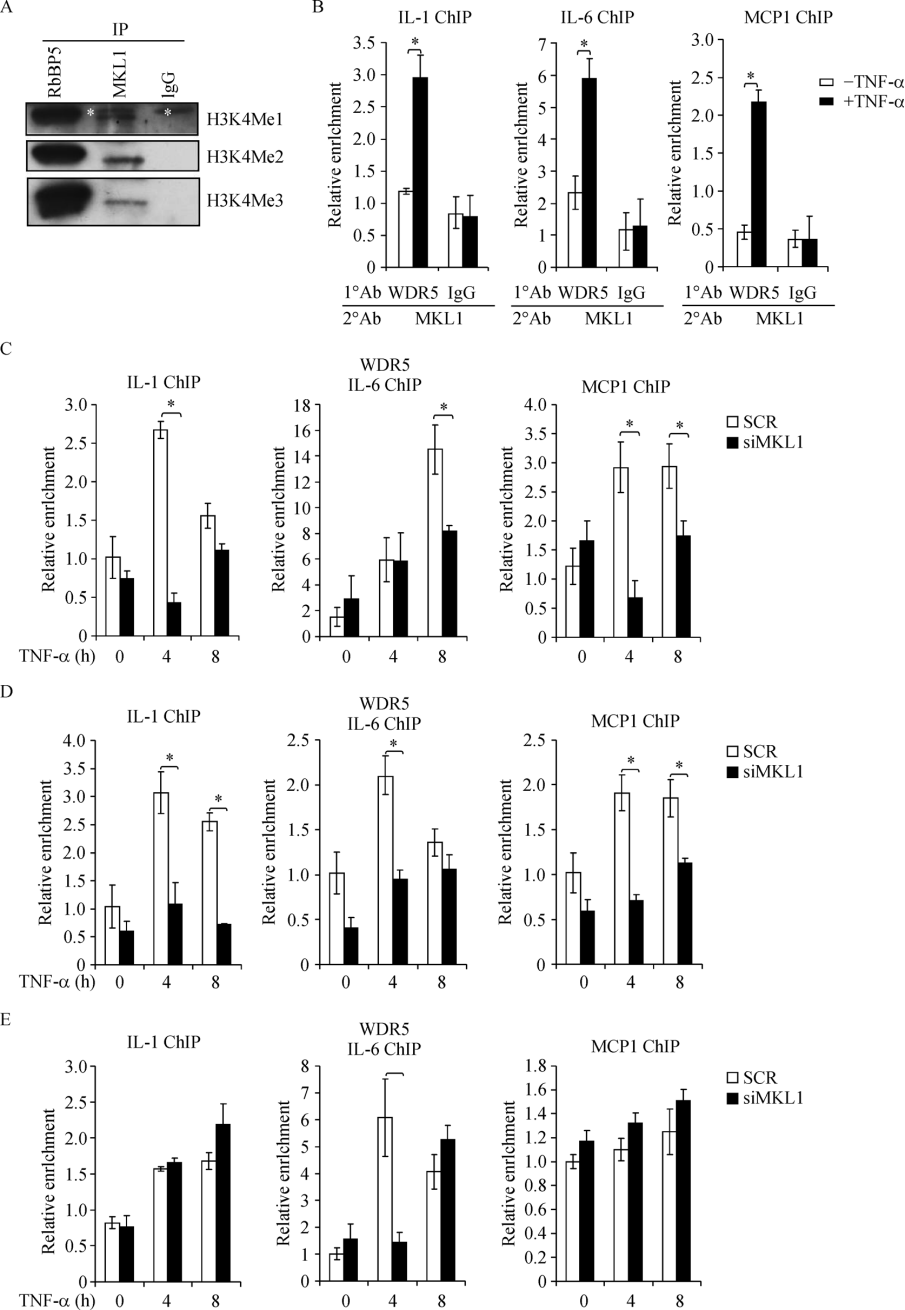
MKL1 is associated with histone H3K4 methyltransferase activity in macrophages. THP-1 cells. HMTassay was performed as described in Methods. White asterisk, non-specific band. B: THP-1 cells were treated with or without TNF-¦Á for 12 hours. Re-ChIP assay was performed with indicated antibodies. C¨CD: THP-1 cells were transfected with siRNA targeting MKL1 or scrambled siRNA (SCR) followed by treatment with TNF-¦Á for 12 hours. ChIP assay was performed with anti-WDR5 (C), anti-BRG1 (D), or anti-Brm (E).

We have previously shown that MKL1 mediates LPS-induced inflammatory response by influencing histone H3K4 methylation^[9]^. We asked whether MKL1 might be in a complex with an catalytically capable H3K4 methyltransferase in macrophages. To this end, we performed *In vitro* histone methyltransferase (HMT) assay. MKL1 was isolated from THP-1 cells by immunoprecipitation and incubated with recombinant histone H3 in the presence of the methyl donor SAM; RbBP5, a known component of the H3K4 methyltransferase complex^[13]^, was included as a positive control whereas pre-immune IgG was included as a negative control. As shown in *Fig. 1A*, MKL1 was co-precipitated with an activity sufficient to catalyze H3K4 methylation. Re-ChIP assay showed that in response to TNF-α stimulation MKL1 was incorporated into a complex with WDR5, a well-defined component of the mammalian H3K4 methyltransferase complex^[23]^, on the promoter regions of several pro-inflammatory mediators (*Fig. 1B*). Indeed, TNF-α treatment enhanced the occupancies of WDR5 on gene promoters in macrophages; MKL1 depletion with siRNA, however, significantly downregulated the binding of WDR5 (*Fig. 1C*), suggesting that MKL1 might play a role in recruiting WDR5 to the promoter regions of pro-inflammatory mediators. Of interest, we also observed that MKL1 was responsible for recruiting BRG1 (*Fig. 1D*), the catalytic subunit of the mammalian chromatin remodeling complex, and, to a lesser extent, BRM (*Fig. 1E*) to the gene promoters. Taken together, we conclude that MKL1 is associated with a histone H3K4 methyltransferase complex that includes WDR5, BRG1, and possibly BRM.

### WDR5 cooperates with MKL1 to activate pro-inflammatory transcription

**Fig.2:**
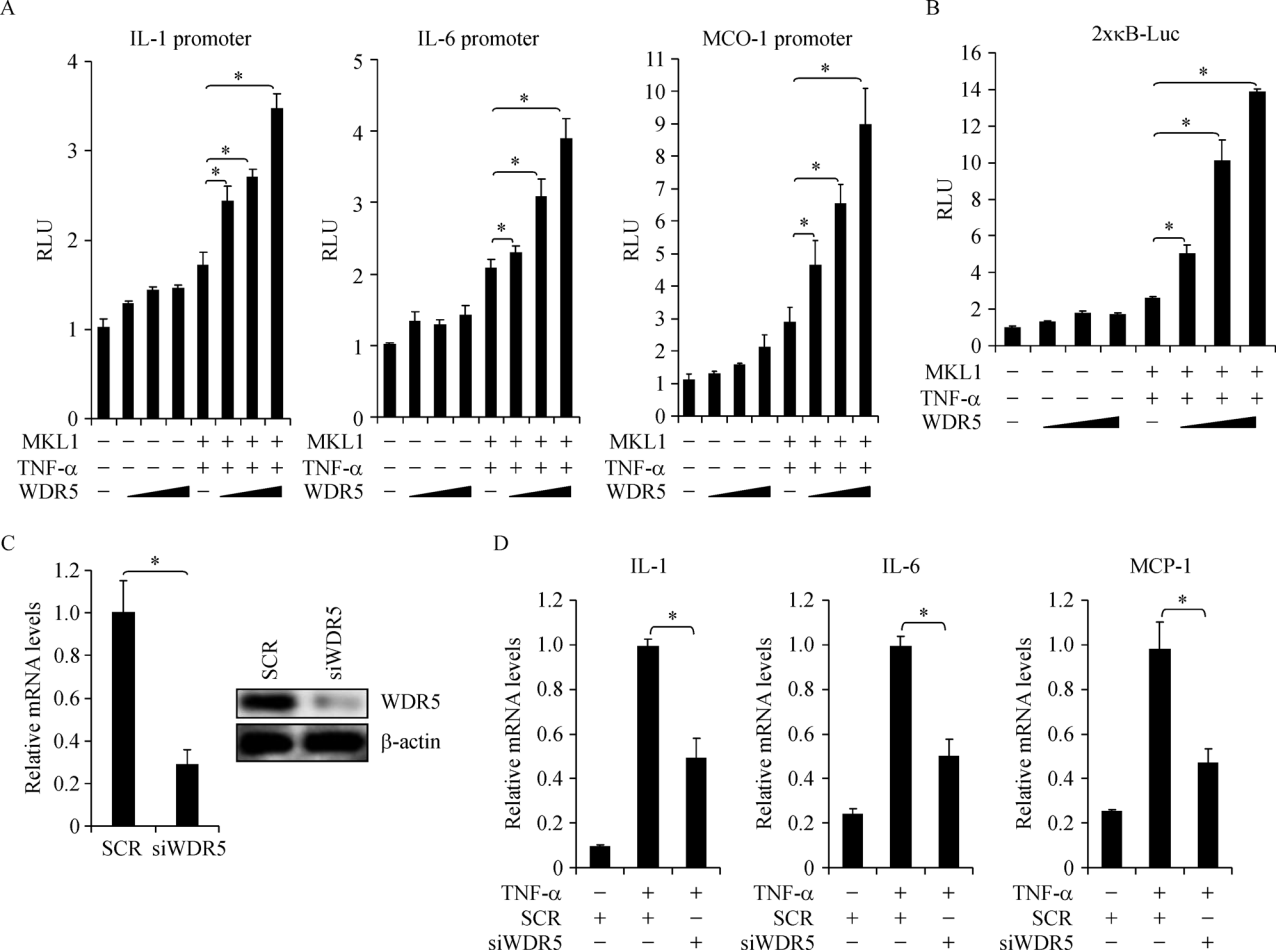
WDR5 cooperates with MKL1 to activate pro-inflammatory transcription. A: Different promoter-luciferase constructs were transfected into HEK293 cells with MKL1 and/or WDR5 followed by treatment with TNF-¦Á for 12 hours. Luciferase activities were normalized by both protein concentration and GFP fluorescence. B: A kB reporter was transfected into HEK293 cells with MKL1 and/or WDR5 followed by treatment with TNF-¦Á for 12 hours. Luciferase activities were normalized by both protein concentration and GFP fluorescence. C: THP-1 cells were transfected with siRNA targeting WDR5 or scrambled siRNA (SCR). Knockdown efficiency was examined by qPCR and Western. D: THP-1 cells were transfected with siRNA targeting WDR5 or scrambled siRNA (SCR) followed by treatment with TNF-¦Á for 12 hours. Expression of pro-inflammatory mediators was examined by qPCR.

Next, we probed the involvement of WDR5 in TNF-α induced trans-activation of pro-inflammatory mediators in macrophages. As shown in *Fig. 2A*, WDR5 was a relatively weak activator of pro-inflammatory genes in reporter assays. In the presence of MKL1, however, WDR5 markedly enhanced the trans-activation of pro-inflammatory genes by TNF-α. Additionally, WDR5 cooperated with MKL1 to upregulate the activity of a κB reporter constructed by fusing two tandem repeats of the NF-κB response element to the luciferase gene (*Fig. 2B*). On the contrary, WDR5 knockdown by siRNA (*Fig. 2C* for efficiency) attenuated the induction of endogenous pro-inflammatory mediators by TNF-α (*Fig. 2D*). Collectively, these data suggest that WDR5 might play a role in mediating the activation of pro-inflammatory genes in response to TNF-α treatment in macrophages.

### WDR5 and BRG1 regulate histone H3K4 methylation surrounding gene promoters

**Fig.3:**
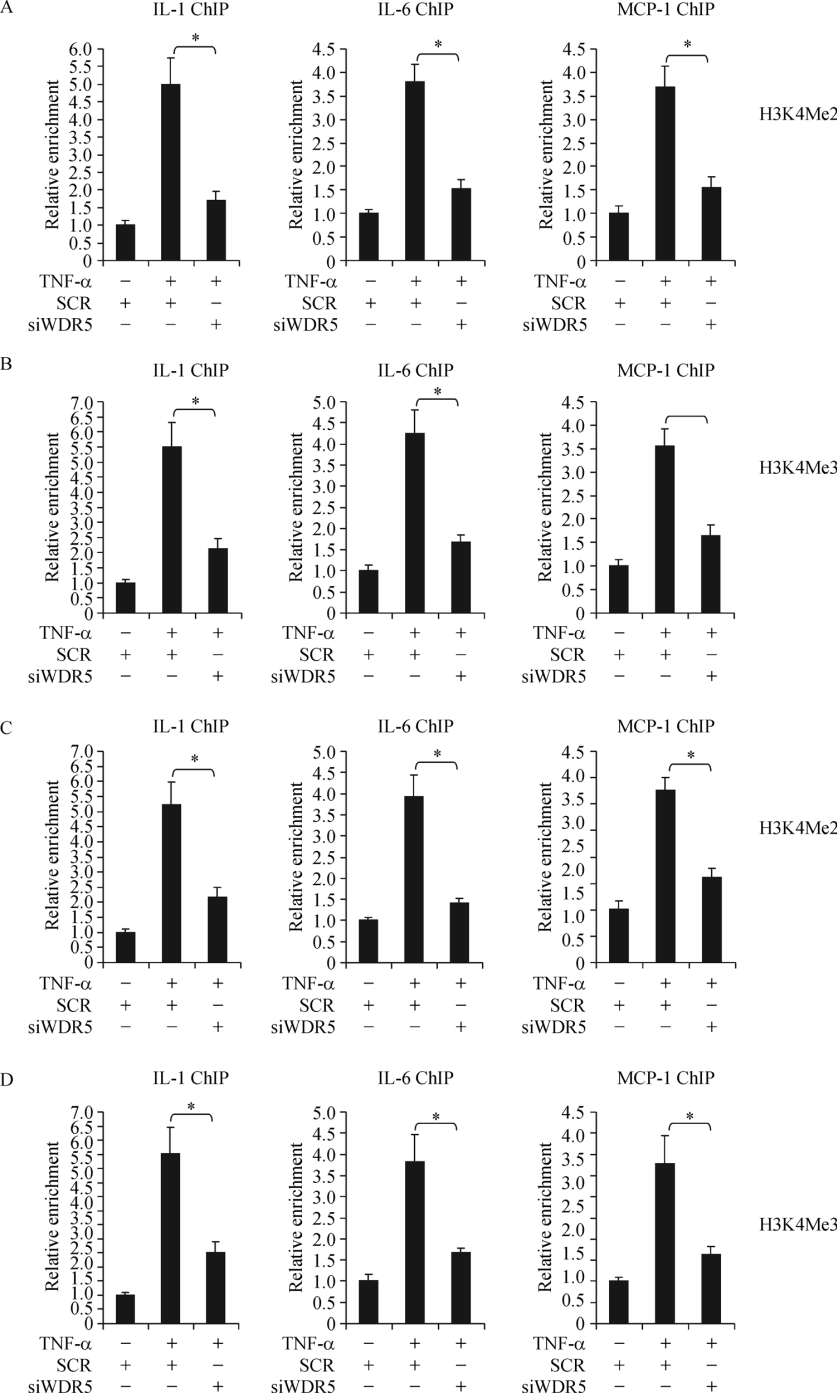
WDR5 and BRG1 regulate histone H3K4 methylation surrounding gene promoters. A¨CB: THP-1 cells were transfected with siRNA targeting WDR5 or scrambled siRNA (SCR) followed by treatment with TNF-¦Á for 12 hours. ChIP assay was performed with anti-H3K4Me2 (A) or anti-H3K4Me3 (B). C¨CD: THP-1 cells were transfected with siRNA targeting BRG1 or scrambled siRNA (SCR) followed by treatment with TNF-¦Á for 12 hours. ChIP assay was performed with anti-H3K4Me2 (C) or anti-H3K4Me3 (D).

**Fig.4:**
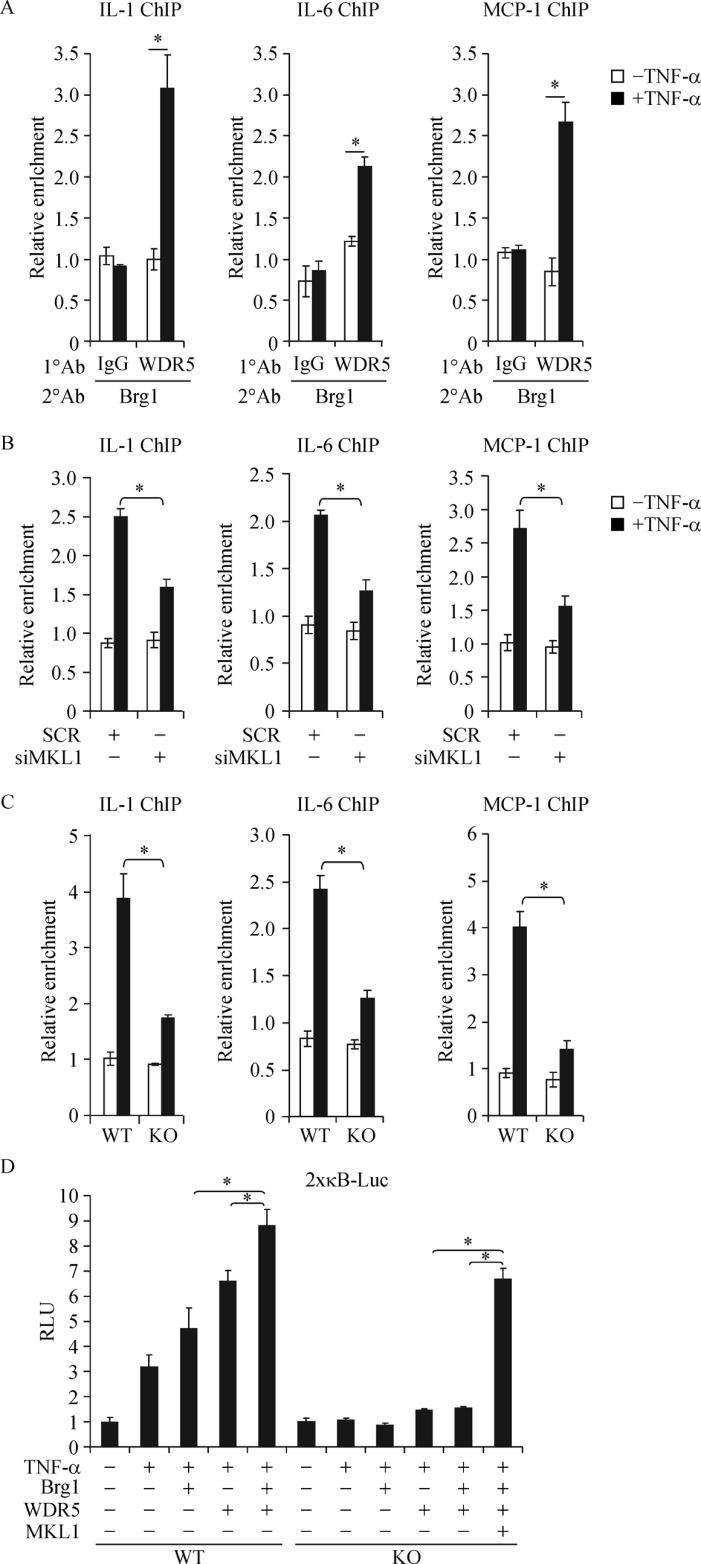
MKL1 mediates the crosstalk between WDR5 and BRG1. A: THP-1 cells were treated with or without TNF-¦Á for 12 hours. Re-ChIP assay was performed with indicated antibodies. B: THP-1 cells were transfected with siRNA targeting MKL1 or scrambled siRNA (SCR) followed by treatment with TNF-¦Á for 12 hours. Re-ChIP assay was performed with indicated antibodies. C: MEF cells isolated from wild type (WT) or MKL1 knockout (KO) mice were treated with or without TNF-¦Á for 12 hours. Re-ChIP assay was performed with indicated antibodies. D: A kB reporter was transfected into WTor KO MEF cells with MKL1, BRG1, and/or WDR5 followed by treatment with TNF-¦Á for 12 hours. Luciferase activities were normalized by both protein concentration and GFP fluorescence.

Next, we tackled the potential mechanism whereby WDR5 contributes to the regulation of pro-inflammatory transcription. Knockdown of WDR5 almost completely erased histone H3K4 di-methylation (*Fig. 3A*) and tri-methylation (*Fig. 3B*), two prominent markers for transcriptional activation^[24]^, surrounding the proximal promoter regions of pro-inflammatory genes. Of note, BRG1 also appeared to play a role in the regulation of histone H3K4 methylation surrounding gene promoters because BRG1 knockdown achieved similar effects as WDR5 knockdown (*Fig. 3C*–*3D*), suggesting of a possible interplay between WDR5 and BRG1 in the regulation of pro-inflammatory transcription. Indeed, Re-ChIP assays showed that WDR5 formed a complex with BRG1 on the proximal promoter regions of pro-inflammatory genes in macrophages treated with TNF-α (*Fig. 4A*). Therefore, WDR5 might regulate TNF-α induced pro-inflammatory transcription by interacting with BRG1 to promote histone H3K4 methylation.

### MKL1 mediates the crosstalk between WDR5 and BRG1

Finally, we asked whether MKL1 could broker the communication between WDR5 and BRG1 to regulate TNF-α induced pro-inflammatory transcription. Depletion of MKL1 with siRNA significantly weakened the interaction between WDR5 an BRG1 on the proximal promoter regions of pro-inflammatory genes (*Fig. 4B*). Similarly, the interaction between WDR5 and BRG1 on the proximal promoter regions of pro-inflammatory genes was much stronger in bone-marrow derived macrophages (BMDM) isolated from wild type (WT) mice than from MKL1 knockout (KO) mice (*Fig. 4C*). Finally, we found that co-expression of WDR5 and BRG1 enhanced the 2xκB reporter activity in WT mouse embryonic fibroblast (MEF) cells but not in KO MEF cells; re-introduction of ectopic MKL1 restored the cooperation between WDR5 and BRG1 (*Fig. 4D*). In summary, we propose that MKL1 could moderate the crosstalk between different epigenetic factors to stimulate TNF-α induced pro-inflammatory transcription.

## Discussion

Epigenetic regulation of gene expression during the pathogenesis of human diseases remains an intriguing and everlasting area of research^[25^–^27]^. We present evidence here that MKL1 plays an essential role in bridging the dialog between BRG1, a chromatin remodeling protein, and WDR5, a subunit of the H3K4 methyltransferase complex, to stimulate the transcription of NF-κB dependent pro-inflammatory transcription. Several independent investigations have implicated BRG1 in the regulation of H3K4 methylation. For instance, Zager and Johnson have shown that during renal ischemia-reperfusion injury, enhanced binding of BRG1 to gene promoters is paralleled by progressive elevations of H3K4 methylation although it remains undetermined whether there is a cause-effect relationship^[28]^. Davis *et al* have reported that treatment of the BRG1 null SW-13 cells with HDAC inhibitors restores BRG1 expression and at the same time stimulates H3K4 hypermethylation^[29]^.We have previously shown that BRG1 is essential in maintaining H3K4 methylation on the endothelin (ET-1) gene promoter, likely through interacting with WDR5, in response to angiotensin II stimulation^[22]^. These data clearly establish a role for BRG1 in regulating locus-specific H3K4 methylation. It is not known at this point how genomewide H3K4 methylation patterns would be influenced by BRG1. In human embryonic stem cells, BRG1 has been found to co-occupy with monomethylated H3K4, catalyzed by the MLL3/4-containing COMPASS complex^[30]^, on early developmental enhancers^[31]^. In cancer cells, loss-of-function mutation of BRG1 appears to influence genomewide distribution of H3K4 trimethylation although the mechanism is not clear^[32]^. Future studies should make an effort in deciphering the connection between BRG1 and H3K4 methylation in a cell- and context-dependent manner.

We show that WDR5 cooperates with BRG1 to stimulate NF-κB dependent pro-inflammatory transcription. Of note, WDR5 has been reported to form extensive communications with other epigenetic factors to regulate transcription. For instance, WDR5 can interact with the acetyltransferase p300 in cancer cells and renal epithelial cells to regulate p53-dependent^[33]^ and SMAD-dependent^[34]^ transcription, respectively. WDR5 can also collaborate with PRMT5, a histone arginine methyltransferase, to activate the transcription of class II major histocompatibility complex (MHC II) genes^[35]^. Of interest, the interaction between WDR5 and GClnc1, a long non-coding RNA (lncRNA), ensures its recruitment to a subset of pro-metastasis genes to activate transcription in gastric cancer cells^[36]^, suggesting that non-protein factors could also contribute to the tethering of WDR5 to selective promoters although it is not clear whether these processes are independent of each other or act in concert. The dynamic interplay between WDR5 and other epigenetic factors likely reflect the extricate nature of transcriptional regulation, which deserves further investigation.

Our data suggest that MKL1 contributes to NF-κB dependent pro-inflammatory transcription by moderating the crosstalk between WDR5 and BRG1. Mounting evidence suggests that MKL1 relies heavily on the epigenetic machinery to regulate transcription^[8^–^10^,^21^–^22^,^34^,^37^–^41]^. In keeping with our finding, Yu *et al*. have demonstrated that MKL1 is responsible for the dissemination of H3K4 trimethylation on the promoter regions of genes involved in inflammation across the genome^[42]^. It remains unanswered whether this function of MKL1 relies on WDR5 and/or BRG1. ChIP-seq analysis of MKL1 in macrophages has found that in addition to NF-κB, MKL1 also preferentially binds to the target promoters of SRF, STAT, and IRF1, all of which are key regulators of inflammatory response^[43]^. It would be interesting to determine whether MKL1 exploits a similar strategy to stimulate the transcription of those target genes.

In summary, we present evidence to show that MKL1 mediates TNF-α induced pro-inflammatory transcription by bridging the crosstalk between BRG1 and WDR5. A recent study has found that small peptides that disrupt the interaction between WDR5 and MLL1/SET1 specifically inhibits the activity of MLL1/SET1-containing COMPASS^[44]^. It is possible to employ this strategy to interfere with the MKL1-WDR5-BRG1 interaction in order to control cellular inflammatory response in an attempt to treat inflammation-related diseases.

## References

[1] Murray PJ, Wynn TA. Protective and pathogenic functions of macrophage subsets[J]. *Nat Rev Immunol*, 2011, 11(11): 723–737. 21997792 10.1038/nri3073 PMC3422549

[2] Chawla A, Nguyen KD, Goh YP. Macrophage-mediated inflammation in metabolic disease[J]. *Nat Rev Immunol*, 2011, 11(11): 738–749. 21984069 10.1038/nri3071 PMC3383854

[3] Moore KJ, Sheedy FJ, Fisher EA. Macrophages in atherosclerosis: a dynamic balance[J]. *Nat Rev Immunol*, 2013,13(10):709–721. 23995626 10.1038/nri3520 PMC4357520

[4] Hernandez C, Huebener P, Schwabe RF. Damage-associated molecular patterns in cancer: a double-edged sword[J]. *Nat Rev Immunol*, 2016, 35(46): 5931–5941 . 27086930 10.1038/onc.2016.104 PMC5119456

[5] Borsig L, Wolf MJ, Roblek M, Inflammatory chemokines and metastasis–tracing the accessory[J]. *Oncogene*, 2014, 33(25):3217–3224. 23851506 10.1038/onc.2013.272

[6] Medzhitov R, Horng T. Transcriptional control of the inflammatory response[J]. *Nat Rev Immunol*, 2009, 9(10):692–703. 19859064 10.1038/nri2634

[7] Fang F, Yang Y, Yuan Z, Myocardin-related transcription factor A mediates OxLDL-induced endothelial injury[J]. *Circ Res*, 2011, 108(7): 797–807 . 21330600 10.1161/CIRCRESAHA.111.240655

[8] Chen D, Yang Y, Cheng X, Megakaryocytic leukemia 1 directs a histone H3 lysine 4 methyltransferase complex to regulate hypoxic pulmonary hypertension[J]. *Hypertension*, 2015,65(4):821–833. 25646298 10.1161/HYPERTENSIONAHA.114.04585

[9] Yu L, Weng X, Liang P, MRTF-A mediates LPS-induced pro-inflammatory transcription by interacting with the COMPASS complex[J]. *J Cell Sci*, 2014, 127(Pt 21): 4645–4657 . 25189621 10.1242/jcs.152314

[10] Yang Y, Cheng X, Tian W, MRTF-A steers an epigenetic complex to activate endothelin-induced pro-inflammatory transcription in vascular smooth muscle cells[J]. *Nucleic Acids Res*, 2014,42(16):10460–10472. 25159611 10.1093/nar/gku776 PMC4176337

[11] Rivera CM, Ren B. Mapping human epigenomes[J]. *Cell*, 2013,155(1):39–55. 24074860 10.1016/j.cell.2013.09.011 PMC3838898

[12] Audia JE, Campbell RM. Histone modifications and cancer[J]. *Cold Spring Harb Perspect Biol*, 2016, 8(4): a019521 . 27037415 10.1101/cshperspect.a019521 PMC4817802

[13] Shilatifard A. The COMPASS family of histone H3K4 methylases: mechanisms of regulation in development and disease pathogenesis[J]. *Annu Rev Biochem*. 2012,81: 65–95. 22663077 10.1146/annurev-biochem-051710-134100 PMC4010150

[14] Ramirez-Carrozzi VR, Nazarian AA, Li CC, Selective and antagonistic functions of SWI/SNF and Mi-2beta nucleosome remodeling complexes during an inflammatory response[J]. *Genes Dev*, 2006, 20(3): 282–296 . 16452502 10.1101/gad.1383206 PMC1361700

[15] Tian W, Xu H, Fang F, Brahma-related gene 1 bridges epigenetic regulation of proinflammatory cytokine production to steatohepatitis in mice[J]. *Hepatology*, 2013, 58(2): 576–588 . 23281043 10.1002/hep.26207

[16] Wang X, Zhu K, Li S, MLL1, a H3K4 methyltransferase, regulates the TNFalpha-stimulated activation of genes downstream of NF-kappaB[J]. *J Cell Sci*, 2012,125(Pt 17): 4058–4066. 22623725 10.1242/jcs.103531

[17] Austenaa L, Barozzi I, Chronowska A, The histone methyltransferase Wbp7 controls macrophage function through GPI glycolipid anchor synthesis[J]. *Immunity*, 2012, 36(4): 572–585. 22483804 10.1016/j.immuni.2012.02.016

[18] El-Osta A, Brasacchio D, Yao D, Transient high glucose causes persistent epigenetic changes and altered gene expression during subsequent normoglycemia[J]. *J Exp Med*, 2008, 205(10): 2409–2417. 18809715 10.1084/jem.20081188 PMC2556800

[19] Sun Y, Boyd K, Xu W, Acute myeloid leukemia-associated Mkl1 (Mrtf-a) is a key regulator of mammary gland function[J]. *Mol Cell Biol*, 2006, 26(15): 5809–5826 . 16847333 10.1128/MCB.00024-06 PMC1592762

[20] Fang F, Chen D, Yu L, Proinflammatory stimuli engage Brahma related gene 1 and Brahma in endothelial injury[J]. *Circ Res*, 2013, 113(8): 986–996 . 23963727 10.1161/CIRCRESAHA.113.301296 PMC4049295

[21] Tian W, Hao C, Fan Z, Myocardin related transcription factor A programs epigenetic activation of hepatic stellate cells[J]. *J Hepatol*, 2015, 62(1): 165–174 . 25109772 10.1016/j.jhep.2014.07.029

[22] Weng X, Yu L, Liang P, A crosstalk between chromatin remodeling and histone H3K4 methyltransferase complexes in endothelial cells regulates angiotensin II-induced cardiac hypertrophy[J]. *J Mol Cell Cardiol*, 2015, 82: 48–58 . 25712920 10.1016/j.yjmcc.2015.02.010

[23] Wysocka J, Swigut T, Milne TA, WDR5 associates with histone H3 methylated at K4 and is essential for H3 K4 methylation and vertebrate development[J]. *Cell*, 2005,121(6):859–872. 15960974 10.1016/j.cell.2005.03.036

[24] Lee JS, Shukla A, Schneider J, Histone crosstalk between H2B monoubiquitination and H3 methylation mediated by COMPASS[J]. *Cell*, 2007, 131(6): 1084–1096. 18083099 10.1016/j.cell.2007.09.046

[25] Xu Y. Transcriptional regulation of endothelial dysfunction in atherosclerosis: an epigenetic perspective[J]. *J Biomed Res*, 2014, 28(1): 47–52 . 24474963 10.7555/JBR.27.20130055 PMC3904174

[26] Xu Y, Fang F. Regulatory role of Brg1 and Brm in the vasculature: from organogenesis to stress-induced cardiovascular disease[J]. *Cardiovasc Hematol Disord Drug Targets*, 2012, 12(2): 141–145. 23030450 10.2174/1871529x11202020141

[27] Xu Y, Fang F. Histone methylation and transcriptional regulation in cardiovascular disease[J]. *Cardiovasc Hematol Disord Drug Targets*, 2014, 14(2): 89–97 . 24801729 10.2174/1871529x14666140505122144

[28] Zager RA, Johnson AC. Renal ischemia-reperfusion injury upregulates histone-modifying enzyme systems and alters histone expression at proinflammatory/profibrotic genes[J]. *Am J Physiol Renal Physiol*, 2009, 296(5): F1032–F1041. 19261745 10.1152/ajprenal.00061.2009 PMC2681356

[29] Davis MR, Daggett JJ, Pascual AS, Epigenetically maintained SW13+ and SW13- subtypes have different oncogenic potential and convert with HDAC1 inhibition[J]. *BMC Cancer*, 2016, 16: 316 . 27188282 10.1186/s12885-016-2353-7 PMC4870788

[30] Sze CC, Shilatifard A. MLL3/MLL4/COMPASS family on epigenetic regulation of enhancer function and cancer[J]. *Cold Spring Harb Perspect Med*, 2016, 6(11): a026427 . 27638352 10.1101/cshperspect.a026427 PMC5088509

[31] Rada-Iglesias A, Bajpai R, Swigut T, A unique chromatin signature uncovers early developmental enhancers in humans[J]. *Nature*, 2011, 470(7333): 279–283 . 21160473 10.1038/nature09692 PMC4445674

[32] Stanton BZ, Hodges C, Calarco JP, Smarca4 ATPase mutations disrupt direct eviction of PRC1 from chromatin[J]. *Nat Genet*, 2017, 49(2): 282–288 . 27941795 10.1038/ng.3735 PMC5373480

[33] Tang Z, Chen WY, Shimada M, SET1 and p300 act synergistically, through coupled histone modifications, in transcriptional activation by p53[J]. *Cell*, 2013, 154(2): 297–310. 23870121 10.1016/j.cell.2013.06.027 PMC4023349

[34] Xu H, Wu X, Qin H, Myocardin-related transcription factor a epigenetically regulates renal fibrosis in diabetic nephropathy[J]. *J Am Soc Nephrol*, 2015, 26(7): 1648–1660 . 25349198 10.1681/ASN.2014070678 PMC4483593

[35] Fan Z, Kong X, Xia J, The arginine methyltransferase PRMT5 regulates CIITA-dependent MHC II transcription[J]. *Biochimica et biophysica acta*, 2016, 1859(5): 687–696. 26972221 10.1016/j.bbagrm.2016.03.004

[36] Sun TT, He J, Liang Q, LncRNA GClnc1 promotes gastric carcinogenesis and may act as a modular scaffold of WDR5 and KAT2A complexes to specify the histone modification pattern[J]. *Cancer Discov*, 2016, 6(7): 784–801 . 27147598 10.1158/2159-8290.CD-15-0921

[37] Tian W, Fan Z, Li J, Myocardin-related transcription factor A (MRTF-A) plays an essential role in hepatic stellate cell activation by epigenetically modulating TGF-β signaling[J]. *Int J Biochem Cell Biol*, 2016, 71: 35–43 . 26693892 10.1016/j.biocel.2015.12.005

[38] Weng X, Yu L, Liang P, Endothelial MRTF-A mediates angiotensin II induced cardiac hypertrophy[J]. *J Mol Cell Cardiol*, 2015, 80(1): 23–33 . 25446178 10.1016/j.yjmcc.2014.11.009

[39] Fan Z, Hao C, Li M, MKL1 is an epigenetic modulator of TGF-beta induced fibrogenesis[J]. *Biochimica et Biophysica Acta*, 2015, 1849(9): 1219–1228. 26241940 10.1016/j.bbagrm.2015.07.013

[40] Cheng X, Yang Y, Fan Z, MKL1 potentiates lung cancer cell migration and invasion by epigenetically activating MMP9 transcription[J]. *Oncogene*, 2015, 34(44): 5570–5581 . 25746000 10.1038/onc.2015.14

[41] Yang Y, Chen D, Yuan Z, Megakaryocytic leukemia 1 (MKL1) ties the epigenetic machinery to hypoxia-induced transactivation of endothelin-1[J]. *Nucleic Acids Res*, 2013, 41(12): 6005–6017 . 23625963 10.1093/nar/gkt311 PMC3695508

[42] Yu L, Fang F, Dai X, MKL1 defines the H3K4Me3 landscape for NF-&B dependent inflammatory response[J]. *Sci Rep*, 2017, 7(1) :191. 28298643 10.1038/s41598-017-00301-w PMC5428227

[43] Xie L. MKL1/2 and ELK4 co-regulate distinct serum response factor (SRF) transcription programs in macrophages[J]. *BMC genomics*, 2014; 15: 301. 24758171 10.1186/1471-2164-15-301 PMC4023608

[44] Alicea-Velázquez NL, Shinsky SA, Loh DM, Targeted disruption of the interaction between WD-40 repeat protein 5 (WDR5) and mixed lineage leukemia (MLL)/SET1 family proteins specifically inhibits MLL1 and SETd1A Methyltransferase Complexes[J]. *J Biol Chem*, 2016, 291(43): 22357–22372 . 27563068 10.1074/jbc.M116.752626 PMC5077178

